# Prognostic role of p27^Kip1^ and apoptosis in human breast cancer

**DOI:** 10.1038/sj.bjc.6690250

**Published:** 1999-03

**Authors:** J Wu, Z-Z Shen, J-S Lu, M Jiang, Q-X Han, J A Fontana, S H Barsky, Z-M Shao

**Affiliations:** Department of Surgery, Molecular Biology Laboratory, Cancer Hospital/Cancer Institute, Shanghai Medical University, 200032, People's Republic of China; University of Maryland Cancer Center, University of Maryland at Baltimore, Baltimore, MD, 21201, USA; Department of Pathology and UCLA/Revlon Breast Center, UCLA, Los Angeles, CA, 90024, USA

**Keywords:** p27^Kip1^, AI, p53, breast cancer, prognosis

## Abstract

Human breast carcinoma is biologically heterogeneous, and its clinical course may vary from an indolent slowly progressive one to a course associated with rapid progression and metastatic spread. It is important to establish prognostic factors which will define subgroups of patients with low vs high risk of recurrence so as to better define the need for additional therapy. Additional characterization of the molecular make-up of breast cancer phenotypes should provide important insights into the biology of breast cancer. In the present study, we investigated apoptosis, expression of p27^Kip1^ and p53 retrospectively in 181 human breast cancer specimens. In addition, their relevance to the biological behaviour of breast cancer was examined. Our studies found a significant association among high histological grade, high p53, low apoptosis and low p27. Our results also demonstrated that, in human breast cancer, low levels of p27 and apoptotic index (AI) strongly correlated with the presence of lymph node metastasis and decreased patient survival. In node-negative patients, however, p27 also had prognostic value for relapse-free and overall survival in multivariate analysis. Furthermore p27 and AI had predictive value for the benefits of chemotherapy. These latter observations should prompt prospective randomized studies designed to investigate the predictive role of p27 and AI in determining who should receive chemotherapy in node-negative patients. © 1999 Cancer Research Campaign


*British Journal of Cancer* (1999) **79**(9/10), 1572–1578
© 1999 Cancer Research Campaign
Article no. bjoc.1998.0250

### Prognostic role of p27$^{Kip1}$ and apoptosis in human breast cancer

**J Wu[1], Z-Z Shen[1], J-S Lu[1], M Jiang[1], Q-X Han[1], JA Fontana[2], SH Barsky[3] and Z-M Shao[1]**

[1]Department of Surgery, Molecular Biology Laboratory, Cancer Hospital/Cancer Institute, Shanghai Medical University, Shanghai, 200032, People's Republic of China; [2]University of Maryland Cancer Center, University of Maryland at Baltimore, Baltimore MD 21201, USA; and [3]Department of Pathology and UCLA/Revlon Breast Center, UCLA, Los Angeles, CA 90024, USA

**Summary** Human breast carcinoma is biologically heterogeneous, and its clinical course may vary from an indolent slowly progressive one to a course associated with rapid progression and metastatic spread. It is important to establish prognostic factors which will define subgroups of patients with low vs high risk of recurrence so as to better define the need for additional therapy. Additional characterization of the molecular make-up of breast cancer phenotypes should provide important insights into the biology of breast cancer. In the present study, we investigated apoptosis, expression of p27$^{Kip1}$ and p53 retrospectively in 181 human breast cancer specimens. In addition, their relevance to the biological behaviour of breast cancer was examined. Our studies found a significant association among high histological grade, high p53, low apoptosis and low p27. Our results also demonstrated that, in human breast cancer, low levels of p27 and apoptotic index (AI) strongly correlated with the presence of lymph node metastasis and decreased patient survival. In node-negative patients, however, p27 also had prognostic value for relapse-free and overall survival in multivariate analysis. Furthermore p27 and AI had predictive value for the benefits of chemotherapy. These latter observations should prompt prospective randomized studies designed to investigate the predictive role of p27 and AI in determining who should receive chemotherapy in node-negative patients.

**Keywords:** p27$^{Kip1}$; AI; p53; breast cancer; prognosis

The staging and therapy of breast cancer patients is currently undergoing an evolution toward breast conservation, limited axillary node dissection (sentinel node biopsy) and more frequent use of both neoadjuvant and adjuvant chemotherapy. In order to support this evolution, better prognostic and predictive markers are needed which can be applied to the primary breast carcinoma. In the present study we investigated apoptosis, p27 and p53 retrospectively in 181 human breast cancer specimens and the relevance of these markers to the biological behaviour of breast cancer.

Cell cycle arrest has been associated with checkpoints regulated by cyclin-dependent kinase (cdk) complexes and their inhibitors (cki's). The cyclin-dependent kinase inhibitor p21$^{Cip1}$ is a critical downstream effector in the p53-specific pathway of growth control in mammalian cells (El-Deiry et al, 1993; Harper et al, 1993; Noda et al, 1994). In a previous study, we have shown that, in human breast cancer, p21 expression is strongly related to cellular differentiation and patient survival (Jiang et al, 1997). Other investigators have observed similar results in lung and colon cancers (El-Deiry et al, 1995; Doglioni et al, 1996; Marchetti et al, 1996). p27$^{Kip1}$, another cyclin-dependent kinase inhibitor, which was more recently cloned (Polyk et al, 1994*a,b*; Toyoshima et al, 1994) has been shown to inhibit the kinase activity of cyclin A-cdk2, cyclin B-cdk2, cyclin D-cdk4 and cyclin E-cdk2, by preventing cdk activation, and thereby precluding cells from entering S-phase (Polyk et al, 1994*a,b*; Toyoshima et al, 1994). p27 protein levels and/or activity are up-regulated by growth inhibitory cytokines including transforming growth factor-β (TGF-β) (Reynisdottir et al, 1995). p27 protein levels also have been associated with density arrest and growth factor deprivation (Gray-Bablin et al, 1997). Loss of p27, a negative cell-cycle regulator, therefore may contribute to oncogenesis and tumour progression (Alessandrini et al, 1997).

Programmed cell death (or apoptosis) represents another critical cellular response to a variety of external stimuli including genotoxic (DNA-damaging) stimuli. The p53 protein is a pivotal component of pathways leading to growth arrest as well as apoptosis, suggesting that the two processes may act in concert (Levine et al, 1997). Many studies have established that DNA damage leads to up-regulation and activation of p53, which can result either in arrest at the $G_1$-S checkpoint, by transcriptional activation of p21$^{Cip}$, or induction of apoptosis. It also has been shown that overexpression of p27 in human breast cancer cells can promote apoptosis (Katayose et al, 1997). Because of the relationships between p27, p53 and apoptosis and their potential regulation of the biology of breast carcinoma cells, we decided to examine the prognostic significance of these markers in human breast cancer.

### MATERIALS AND METHODS

### Samples

Breast cancer specimens were obtained from 181 patients. Histological types were determined according to the WHO criteria. Samples were obtained between 1986 and 1990 from patients at the Department of Surgery, Cancer Hospital of Shanghai Medical University following an approved institutional review board protocol.

*Received 23 April 1998*
*Revised 11 September 1998*
*Accepted 29 September 1998*

*Correspondence to:* Z-M Shao

**Table 1** Characteristics of breast cancer patients in study

| Characteristic | Number (*n* = 181) (%) |
|---|---|
| Menopause | |
| Premenopausal | 46.4 |
| Postmenopausal | 53.6 |
| Tumour size | |
| ≤ 2 cm | 30.9 |
| > 2 cm | 69.1 |
| TNM Stage | |
| 1 | 18.2 |
| 2 | 55.2 |
| 3 | 26.6 |
| LN status | |
| LN(–) | 46.4 |
| LN(+) | 53.6 |
| Grade | |
| 1 | 47.5 |
| 2 | 33.1 |
| 3 | 19.4 |
| ER status | |
| ER(+) | 43.6 |
| ER(–) | 56.4 |
| p27 | |
| Low | 69.1 |
| High | 30.9 |
| p53 | |
| Low | 65.0 |
| High | 35.0 |
| AI | |
| Low | 61.3 |
| High | 38.7 |
| Disease status | |
| Relapses | 28.7 |
| No relapses | 71.3 |
| Survival status | |
| Dead | 11 |
| Alive | 89 |
| Therapy | |
| Surgery + chemotherapy | 71.3 |
| Surgery only | 28.7 |

A

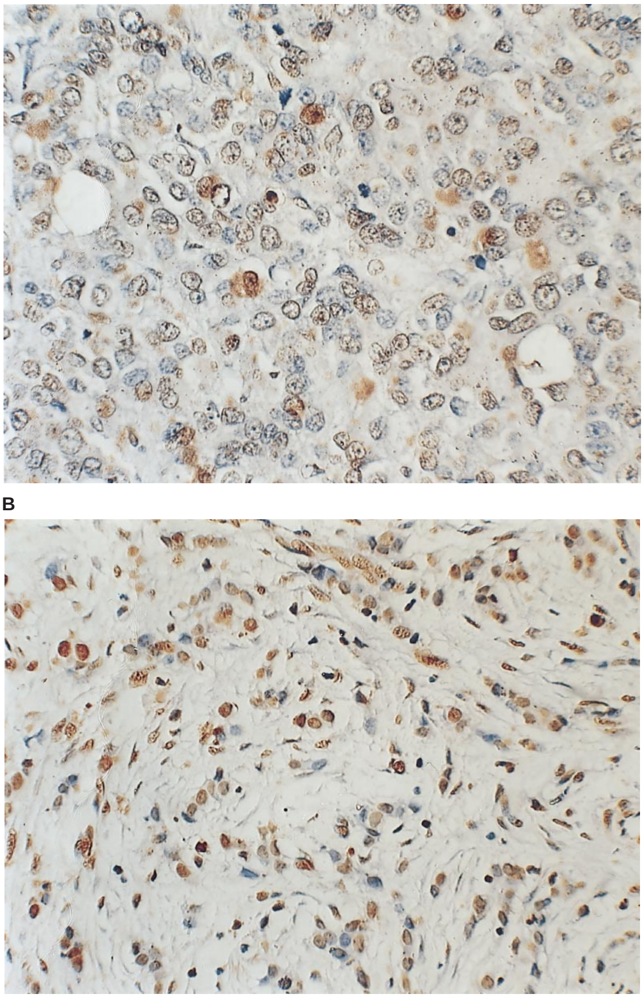

B

**Figure 1** p27 and AI in two infiltrating ductal carcinomas. (**A**) Apoptotic cells (brown nuclear staining) comprised 5% of all cells in this case whose AI would fall in the AI (H) category, magnification × 250. **(B)** p27 immunopositive (brown nuclear staining) cells comprise approximately 60% of all cells in this case whose p27 would fall in the p27 (H) category, magnification × 100

### Immunohistochemical analysis

Immunohistochemical analysis of p27 and p53 protein expression in breast carcinoma samples was performed. Paraffin sections (5 μm) obtained from biopsies were subjected to immunoperoxidase staining with murine monoclonal antibodies: anti-human p27 (PharMingen, San Diego, CA), and anti-human p53 (Oncogene Research, Cambridge, MA 02142). Tissue sections were deparaffinized with two changes of xylene for 5 min, followed by two washes of absolute ethanol, 95% and 70% ethanol for 3 min each and then treated with 2% $H_2O_2$ in methanol for 30 min to quench endogenous peroxidase. The sections were blocked with diluted goat serum for 30 min. The sections were incubated with 50 μl monoclonal mouse anti-human p27 (dilution of 1:50) or p53 antibody (dilution of 1:50) at room temperature for 1 h. Control sections were incubated in the absence of primary antibody. The sections were washed and then incubated with diluted (1:200) biotinylated goat anti-mouse IgG for 1 h at room temperature, followed by a 60-min incubation with ABC reagent. Staining was developed using 3,3′-diaminobenzidine for varying times to optimize target/background staining. Cells were considered positive for p27 and p53 when distinct nuclear staining was identified. Representative areas of the tumour, i.e. areas in which staining was most interpretable, were randomly chosen for study. The percentage of cells demonstrating positive nuclear staining was evaluated by counting 1000 cells in random high power fields. Two observers counted both markers. Interobserver variation was addressed by averaging the individual values. Interobserver variation usually did not differ by more than 10%.

### Apoptosis assay

Apoptosis was detected by labelling the 3’ OH ends of DNA utilizing digoxigenin-nucleotide incorporation by terminal deoxynucleotidyl transferase, a type of TUNEL method. Antidigoxigenin antibodies and immunoperoxidase staining were utilized with the ApopTag detection system (Oncor, Gaithersburg, MD, USA). All sections were coded and scored blindly by two

© Cancer Research Campaign 1999

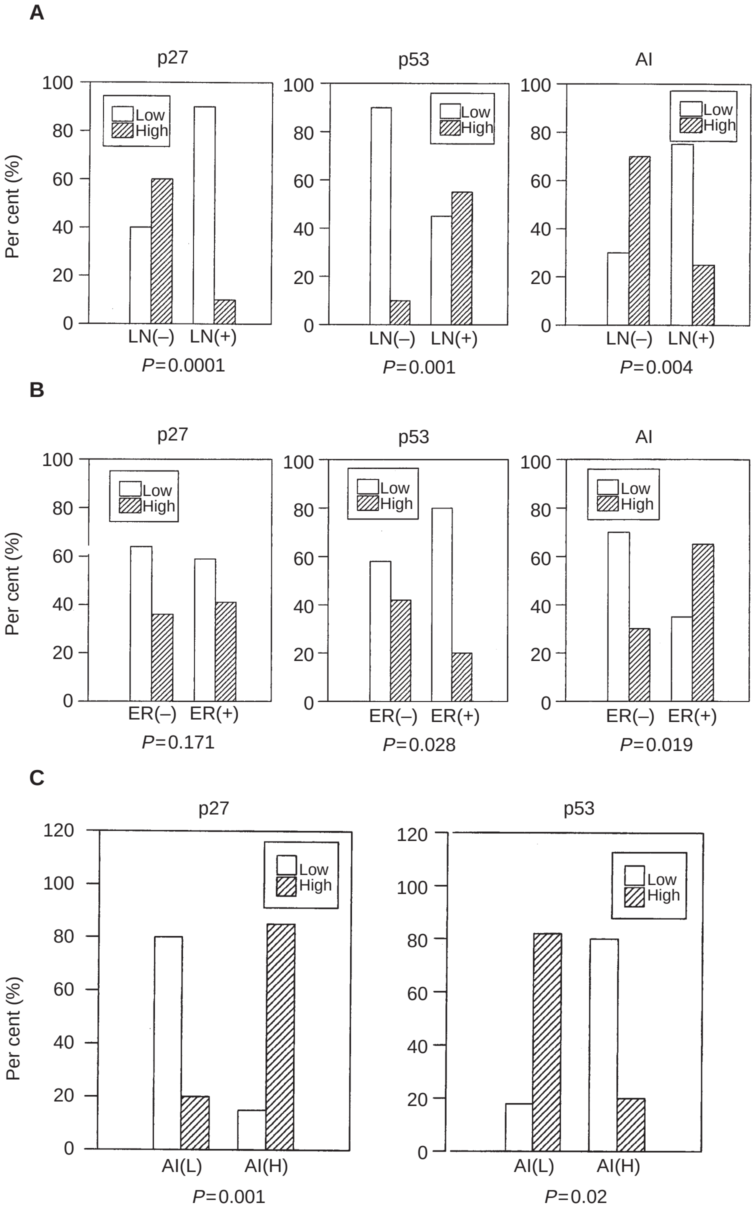

**Figure 2** **(A)** The relationship between p27, p53 and AI in breast cancer samples and axillary lymph node metastasis. **(B)** The relationship between p27, p53 and AI with ER status in breast cancer samples. **(C)** The relationship between p27, p53 and AI

observers in an identical manner as was done for p27 and p53. The percentage of cells showing positive nuclear staining was determined as the apoptotic index (AI).

### Steroid receptor assays

The standard dextran coated-charcoal assay was used as described previously (Kute et al, 1992). In all cases, the Scatchard plot analysis was done with eight points, and the protein content in the reaction was 1 mg $ml^{-1}$. Receptor levels of 10 fmol $mg^{-1}$ of protein or greater were considered positive. The biochemical measurements were subsequently confirmed by ER immunohistochemistry. We used the biochemical determinations of ER in the final analysis because they were more quantitative.

### Statistical analysis

Comparisons of the differences among the expression of p27, p53 and AI were made using the two tailed Student's *t*-test. Spearman's rank-based correlation was used to assess the relationship between the variables. The Kaplan–Meier method was utilized to estimate relapse-free and overall survival times. Log-rank test was used to assess the univariate effect of the expression of p27, 53, AI and other variables on the relapse-free and overall survival times.

*© Cancer Research Campaign 1999*

**Table 2** Correlation between histological grade of breast cancer and p27, p53 and AI

| | Histological grade | |
|---|---|---|
| | *Rs*[a] | *P* |
| p27 | –0.303 | 0.002 |
| p53 | +0.454 | 0.004 |
| AI | –0.479 | 0.0001 |

[a]Spearman test: *R*s: Correlation coefficient.

**Table 3** Patient survival status in different phenotypes of p27, p53 and AI

| Phenotype | Alive and well (%) | Relapse (%) | Death (%) |
|---|---|---|---|
| p27(H) AI(H) | 100 | 0 | 0 |
| p27(L) AI(L) | 52 | 24 | 24 |
| p27(L) AI(H) | 80 | 14 | 6 |
| p27(H) AI(L) | 89 | 11 | 0 |
| p27(H) p53(L) | 93 | 7 | 0 |
| p27(L) p53(H) | 45 | 33 | 22 |
| p27(H) p53(H) | 75 | 0 | 25 |
| p27(L) p53(L) | 71 | 24 | 5 |

H, high expression; L, low expression.

**Table 4** Univariate analysis for prognostic factors for breast cancer patients

| | *P*-values | |
|---|---|---|
| Factors analysis | Relapse-free survival | Overall survival |
| LN status | 0.0032 | 0.0024 |
| Tumour size | 0.0011 | 0.0931 |
| ER status | 0.1709 | 0.0040 |
| p27 | 0.0001 | 0.0012 |
| p53 | 0.0252 | 0.0431 |
| AI | 0.0095 | 0.0341 |

**Table 5** Multivariate analysis with Cox's proportional hazards model for prognostic factors for breast cancer patients

| Factors | Relapse-free survival | | Overall survival | |
|---|---|---|---|---|
| analysis | Risk ratio (CI) | *P*-value | Risk ratio (CI) | *P*-value |
| Age | 1.0875 (0.987–1.169) | 0.1826 | 1.2553 (1.163–1.361) | 0.3393 |
| LN status | 2.2939 (2.218–2.376) | 0.0018 | 2.3701 (2.202–2.398) | 0.0197 |
| Tumour size | 1.2345 (1.171–1.391) | 0.3577 | 1.0761 (0.982–1.165) | 0.7961 |
| ER status | 0.8314 (0.769–0.893) | 0.6341 | 0.6352 (0.539–0.735) | 0.2766 |
| p27 | 0.4132 (0.323–0.514) | 0.042 | 0.3691 (0.252–0.451) | 0.0495 |
| p53 | 0.3317 (0.258–0.417) | 0.0332 | 0.3439 (0.241–0.436) | 0.0465 |
| AI | 0.7235 (0.639–0.807) | 0.3411 | 0.8773 (0.787–0.962) | 0.9385 |

**Table 6** Multivariate analysis of relapse-free and overall survival in lymph node-negative patients

| Factors | Relapse-free survival | | Overall survival | |
|---|---|---|---|---|
| | Risk ratio (CI) | *P*-value | Risk ratio (CI) | *P*-value |
| p27 | 0.1734 (0.054–0.0586) | 0.003 | 0.2646 (0.070–0.996) | 0.049 |
| p53 | 0.2891 (0.056–0.098) | 0.023 | 0.2561 (0.078–0.981) | 0.032 |
| Grade | 3.5461 (1.124–11.19) | 0.031 | 2.555 (0.524–12.46) | 0.246 |
| TNM stage | 5.1691 (0.582–31.37) | 0.074 | 3.9162 (0.591–25.93) | 0.157 |
| ER status | 0.8723 (0.719–0.987) | 0.817 | 0.7343 (0.572–0.896) | 0.633 |
| AI | 1.3561 (1.231–2.345) | 0.341 | 0.8911 (0.761–1.234) | 0.691 |

**Table 7** Multivariate analysis of relapse-free and overall survival in lymph node-positive patients

| Factors | Relapse-free survival | | Overall survival | |
|---|---|---|---|---|
| | Risk ratio (CI) | *P*-value | Risk ratio (CI) | *P*-value |
| p27 | 0.4567 (0.233–0.895) | 0.022 | 0.1849 (0.044–0.769) | 0.020 |
| p53 | 0.2891 (0.156–0.598) | 0.033 | 0.3561 (0.278–0.681) | 0.041 |
| Grade | 2.5091 (1.384–4.547) | 0.002 | 3.4061 (1.319–8.797) | 0.011 |
| TNM stage | 1.549 (0.781–3.069) | 0.210 | 1.204 (0.479–3.025) | 0.693 |
| ER status | 4.331 (1.895–9.898) | <0.001 | 2.036 (0.5423–7.643) | 0.292 |
| AI | 2.3561 (1.231–3.345) | 0.841 | 1.8911 (1.761–2.234) | 1.691 |

Cox's proportional hazards model was used to examine the differences in overall survival and relapse-free survival after adjustment for other covariates.

### RESULTS

The 181 breast cancer patients whose specimens were collected in 1986–1990 were followed-up for periods up to 12 years; the median follow-up time was 5 years. Table 1 gives the characteristics of the patients and their tumours. We used the log-rank test to assign divisions providing the best prognostic separations of our patient database. We defined low AI (L) as those tumours with an AI ≤ 2.10% and high AI (H) > 2.10% (Figure 1A). We found that 38.7% of the tumours showed AI (H) and 61.3% had AI (L). We defined low p27 as tumours in which ≤ 50% of the cells showed nuclear staining and high p27 as tumours in which > 50% of the cells showed nuclear staining (Figure 1B). We found that 30.9% of the tumours showed p27 (H) and 69.1% showed p27 (L). In this study we chose to assign no significance to p27 cytoplasmic staining which was present occasionally. With respect to p53 we defined tumours with ≤ 15% of the cells immunoreactive as low p53 and tumours with > 15% of the cells immunoreactive as high p53. With this division 65.0% of the tumours were p53 (L) and 35.0% were p53 (H). In this study we deviated from the traditional cutoff of 10% for p53 because by log-rank test analysis, 15% provided the best prognostic separation of our patient population.

Our results demonstrated that low p27, low AI and high p53 significantly correlated with lymph node metastasis (Figure 2A). When the relationship between p27, AI and p53 and ER expression was examined, p53 inversely correlated, AI directly correlated, and p27 did not correlate with ER positivity (Figure 2B). Furthermore, we found an inverse relationship between p27 and p53. AI also inversely correlated with p53 expression, but directly correlated with p27 expression (Figure 2C). A significant association was also seen between low p27, low AI, high p53 and high histological grade (Table 2).

On the basis of stratifying our patients into p27/p53 (L vs H) and p27/AI (L vs H) phenotypes, we examined their overall survival and relapse rates. p27 (L)/AI (L) and p27 (L)/p53 (H) exhibited high relapse and death rates (48–55%) ($P < 0.05$) (Table 3).

© Cancer Research Campaign 1999

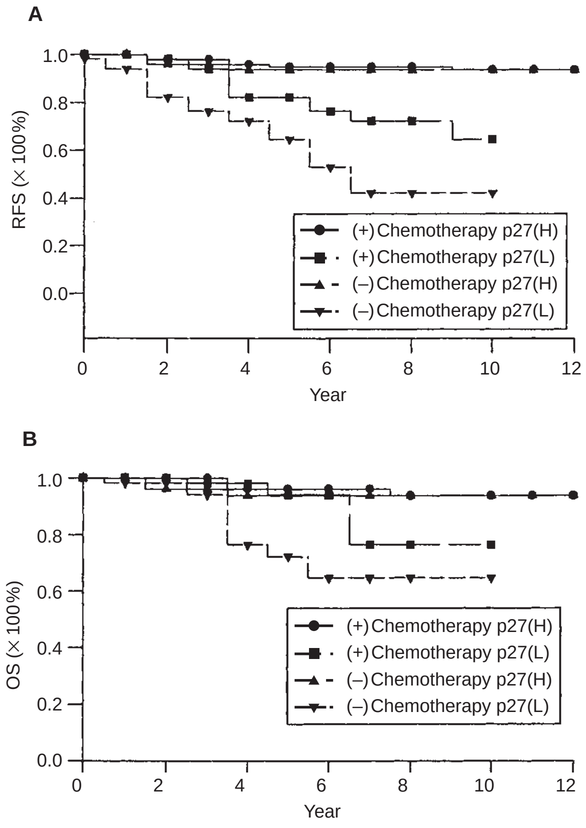

**Figure 3** The (**A**) overall survival and (**B**) relapse-free survival curves in breast cancer patients with low and high p27 who received and did not receive chemotherapy

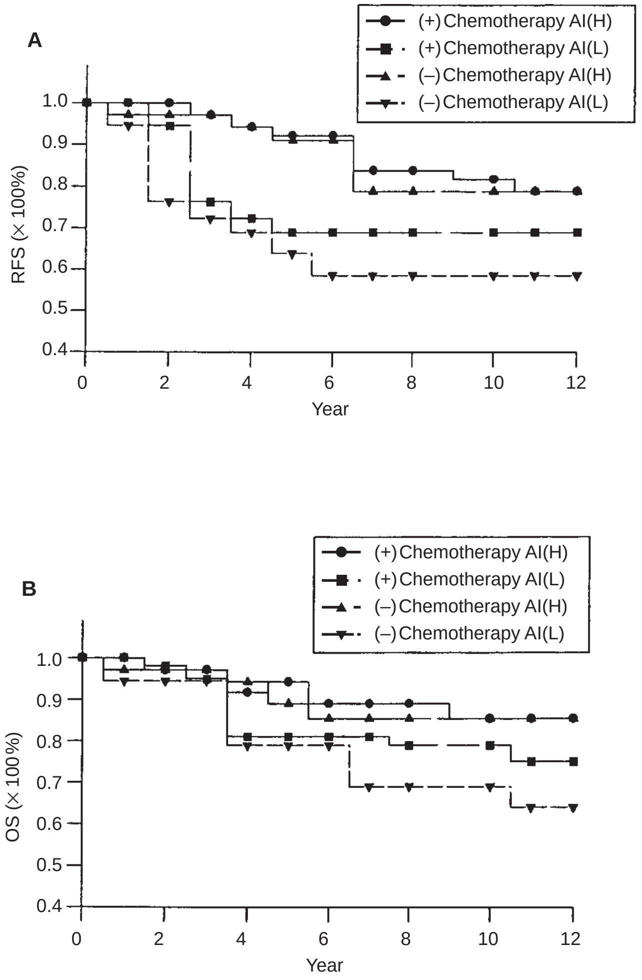

**Figure 4** The (**A**) overall survival and (**B**) relapse-free survival curves in breast cancer patients with low and high AI who received and did not receive chemotherapy

The univariate relationships between p27, p53, AI and traditional tumour characteristics and relapse-free and overall survival are given in Table 4. As shown in this table, p27, AI, p53 and lymph node status were significant prognostic factors for both relapse-free and overall survival.

Cox's proportional hazard model was used to assess the importance of the p27, p53, AI and traditional tumour characteristics in a multivariate analysis (Table 5). Lymph node status, p27 and p53 were significantly predictive of both relapse-free and overall survival, while AI, ER status and age did not prove to be independent prognostic markers. In a subsequent breakdown of this analysis into lymph node-negative (Table 6) and lymph node-positive (Table 7) patients, both p27 and p53 were independent prognostic markers of both relapse-free and overall survival.

When p27 and AI status were examined separately in groups of patients who received or did not receive chemotherapy (including tamoxifen) and related to relapse-free and overall survival, low p27 and low AI were predictive of maximum benefit of chemotherapy ($P < 0.05$) (Figures 3 and 4).

### DISCUSSION

Human breast carcinoma is biologically heterogeneous, and its clinical course may vary from an indolent slowly progressive one to a course associated with rapid progression and metastatic spread. Clinical parameters such as tumour size and lymph node status have long been used to characterize breast cancer phenotypes in relation to prognosis (Saez et al, 1988). It is important, however, especially in node-negative patients, to establish prognostic and predictive factors which will define subgroups of patients. As the staging and therapy of breast cancer patients is currently undergoing an evolution toward breast conservation, more limited axillary node dissection (sentinel node sampling) and more frequent use of both neoadjuvant and adjuvant chemotherapy, it is even more imperative to define better prognostic and predictive markers that can be applied to the actual primary breast cancer tissue specimen (Robertson et al, 1997). In the present study, we investigated apoptosis, expression of $p27^{Kip1}$ and p53 for their prognostic and predictive significance.

It seems logical that an analysis of cell proliferation and cell death in human breast cancer would result in prognostic and predictive markers. Normal mammary epithelial homeostasis is dependent not only on the rate of cell proliferation but also on apoptosis – a genetically programmed process of autonomous cell death (Geske et al, 1994). Defective regulation of apoptosis and proliferation may exert an important effect in breast cancer. Reduced apoptosis may lead to a shift in tissue kinetics towards the expansion of cell numbers, and also to the preservation of

© Cancer Research Campaign 1999

genetically aberrant cells, favouring neoplastic development. In a previous study, we have shown breast cancer cells exhibit reduced apoptosis compared to normal breast epithelium (Shao et al, 1996). Investigators in another study reported reduced breast epithelial cell apoptosis in association with fibrocystic disease and an increased risk of carcinoma (Allan et al, 1992). Other investigators have shown that the inhibition of apoptosis is linked to tumour promotion (Tomei et al, 1988). Higher apoptotic counts have also been observed to be associated with a better outcome in neuroblastoma (Hoehner et al, 1995) and colon cancer (Langlois et al, 1997). In this study, we have demonstrated that high AI was associated with lower tumour grade and lack of axillary lymph node metastasis. In univariate analysis, high AI correlated with increased relapse-free and overall survival. Although multivariate analysis did not show AI as an independent prognostic marker, our results suggest that AI at least partially influences clinical outcome. Low AI was predictive of chemotherapy benefit. Our results can be explained by the hypothesis that cells with low apoptosis have an increased propensity for metastatic survival (Raff et al, 1992) but greater susceptibility to the apoptosis-inducing effects of chemotherapy.

Cell proliferation is governed by the synthesis and activation of a number of positive and negative regulators of cell cycle progression (Hartwell et al, 1994). A number of cyclin/CDK inhibitors have been found associated with the $G_1$ cell cycle period (Hunt et al, 1993; Wage et al, 1994). One of these inhibitors, $p27^{Kip1}$, was identified by a number of investigators independently and appears to play a major role in the regulation of cyclin CDK complex activity in this phase of the cell cycle (Polyk et al, 1994*a,b*; Toyoshima et al, 1994). Overexpression of p27 in mammalian cells induces a G1 block of the cell cycle and inhibits growth of a number of cancer cells (Polyk et al, 1994*a,b*; Toyoshima et al, 1994). p27 immunoreactivity, however, has been investigated in only a limited number of human cancers (Catzavelos et al, 1997; Esposito et al, 1997; Loda et al, 1997; Porter et al, 1997; Tan et al, 1997). Low p27 expression in colon cancer has been related to aggressiveness and decreased survival (Loda et al, 1997). In human breast cancer, low p27 is associated with tumour progression (Catzavelos et al, 1997). In small invasive breast cancers, p27 expression was found to be an independent prognostic marker (Tan et al, 1997). It has also been shown that the expression of p27 and cyclin E, alone and in combination, correlates with survival in young breast cancer patients (Porter et al, 1997). In our present study, we have demonstrated that low p27 strongly correlates with lymph node metastasis while high p27 correlates with improved patient survival. In node-negative patients where there is a great need for prognostic markers, our study demonstrated that p27 is an independent prognostic marker in multivariate analysis. Furthermore, p27 was predictive of chemotherapy benefit.

As p27 belongs to the same family of cki's as p21, and p21 is known to be a downstream effector of p53, there could potentially be a similar relationship between p27 and p53. In our study, we, in fact, found evidence of this downstream relationship. We found an inverse relationship between p27 and p53 expression in human breast carcinomas: low p27 expression was associated with p53 overexpression, a marker of p53 mutational inactivation, while high p27 expression was associated with normal p53 expression. It has been shown that overexpression of $p27^{Kip1}$ in cancer cells results in $G_1$-S arrest and induction of apoptosis (Katayose et al, 1997). In our study, we found that p27 expression strongly correlated with AI ($P < 0.05$).

In the present study, we stratified our patients into p27/AI and p27/p53 phenotypes (L vs H) and examined their relationship to clinical outcome. We found that p27(L)/AI(L) and p27(L)/p53(H) status had the worst prognosis. It is interesting that the p27(L)/p53(L) phenotype also had a particularly high relapse rate. As low p53 could reflect not only normal or wild-type p53 expression but absence of p53 expression (from homozygous mutations or deletions), p53 of the latter categories could explain this phenotype.

In conclusion, our studies show that p27 and AI are potentially important prognostic and predictive markers of outcome in breast cancer patients. It must be remembered, however, that these conclusions must be tempered with the knowledge that our study was a retrospective one. A prospective randomized study is very much needed to examine these results more thoroughly, and presently we are in the process of conducting such a study.

### ACKNOWLEDGEMENTS

This research was supported in part by the National Natural Science Foundation of China (39670803) and the Mrs. Xia Li-Jun Research Foundation.

### ABBREVIATIONS

ER, oestrogen receptor; TUNEL, terminal transferase (TdT)-mediated dUTP nick end-labelling; AI, apoptotic index; CKI, cyclin-dependent kinase inhibitor; CDK, cyclin dependent kinase.

### REFERENCES

Allan DJ, Howell A, Roberts SA, Williams GT, Watson RJ, Coyne JD, Clarke RB, Laidlaw IJ and Potten CS (1992) Reduction in apoptosis relative to mitosis in histologically normal epithelium accompanies fibrocystic change and carcinoma of the premenopausal human breast. *J Pathol* **167**: 25–32

Alessandrini A, Chiaur DS and Pagano M (1997) Regulation of the cyclin-dependent kinase inhibitor p27 by degradation and phosphorylation. *Leukemia* **11**: 342–345

Catzavelos C, Bhattacharya N, Ung YC, Wilson JA, Roncari L, Sandhu C, Shaw P, Yeger H, Morava-Protzner I, Kapusta L (1997) Decreased levels of the cell-cycle inhibitor p27Kip1 protein: prognostic implications in primary breast cancer. *Nature Medicine* **3**: 227–230

Doglioni C, Pelosio P, Laurino L, Macri E, Meggiolaro E, Favretti F and Barbraeschi M (1996) p21/WAF1/CIP1 expression in normal mucosa and in adenomas and adenocarcinomas of the colon: its relationship with differentiation. *J Pathol* **179**: 248–253

El-Deiry WS, Tokino T, Veiculescu VE, Levy DB, Parsons R, Trent LM, Lin D, Mercer WE, Kinzler KW and Vogelstein B (1993) WAF1, a potential mediator of p53 tumor suppression. *Cell* **75**: 817–825

El-Deiry WS, Tokino T and Waldman T (1995) Topological control of p21WAF1/CIP1 expression in normal and neoplastic tissues. *Cancer Res* **55**: 2910–2919

Esposito V, Baldi A, De Luca A, Groger AM, Loda M, Giordano GG, Caputi M, Baldi F, Pagano M and Giordano A (1997) Prognostic role of the cyclin-dependent kinase inhibitor p27 in non-small cell lung cancer. *Cancer Res* **57**: 3381–3385

Geske FJ, Bemis LT, Schedin PJ and Strange R (1994) Function evaluation of genes associated with apoptotic cell death of mammary epithelium. *Proc Annu Meet Am Assoc Cancer Res* **35**: 19–25

Gray-Bablin J, Rao S and Keyomarsi K (1997) Lovastatin induction of cyclin-dependent kinase inhibitors in human breast cells occurs in a cell cycle-independent fashion. *Cancer Res* **57**: 604–609

Harper JW, Adami GR, Wei N, Keyomarsi K and Elledge ST (1993) The p21 CDK-interacting protein Cip1 is a potent inhibitor of G1 cyclin-dependent kinases. *Cell* **75**: 805–816

Hartwell L and Kastan MB (1994) Cell cycle control and cancer. *Science* **266**: 1820–1828

Hoehner JC, Hedborg F, Wiklund HJ, Olsen L and Pahlman S (1995) Cellular death in neuroblastoma: in situ correlation of apoptosis and bcl-2 expression. *Int J Cancer* **62**: 19–24
Hunt T and Nasmyth K (1993) Dams and sluices. *Nature* (Lond) **366**: 634–635
Jiang M, Shao ZM, Wu J, Lu JS, Yu LM, Yuan JD, Han QX, Shen ZZ and Fontana JA (1997) p21/waf1/cip1 and mdm-2 expression in breast carcinoma patients as related to prognosis. *Int J Cancer* **74**: 529–534
Katayose Y, Kim M, Rakkar AN, Li Z, Cowan KH and Seth P (1997) Promoting apoptosis: a novel activity associated with the cyclin-dependent kinase inhibitor p27. *Cancer Res* **57**: 5441–5445
Kute TE, Shao ZM, Sugg NK, Long RT, Russell GB, Case LD (1992) Cathepsin D as a prognostic indicator for node-negative breast cancer patients using both immunoassays and enzymatic assays. *Cancer Res* **52**: 5198–5203
Langlois NE, Lamb J, Eremin O and Heys SD (1997) Apoptosis in colorectal carcinoma occurring in patients aged 45 years and under: relationship to prognosis, mitosis, and immunohistochemical demonstration of p53, c-myc and bcl-2 protein products. *J Pathol* **182**: 392–397
Levine AJ (1997) p53, the cellular gatekeeper for growth and division. *Cell* **88**: 323–331
Loda M, Cukor B, Tam SW, Lavin P, Fiorentino M, Draetta GF, Jessup JM and Pagano M (1997) Increased proteasome-dependent degradation of the cyclin-dependent kinase inhibitor p27 in aggressive colorectal carcinomas. *Nature Med* **3**: 231–234
Marchetti A, Doglioni C and Barbareschi M (1996) p21 mRNA and protein expression in non-small cell lung cancer: evidence of p53-independent expression and association with tumoral differentiation. *Oncogene* **12**: 1319–1324
Noda A, Ning Y, Venable SF, Pereira-Smith OM and Smith JR (1994) Clonation of senescent cell-derived inhibitors of DNA synthesis using an expression screen. *Exp Cell Res* **211**: 90–98
Polyak K, Lee MH, Erdjument-Bromage H, Koff A, Roberts JM, Tempst P and Massague J (1994*a*) Cloning of p27Kip1, a cyclin-dependent kinase inhibitor and a potential mediator of extracellular antimitogenic signals. *Cell* **78**: 59–66
Polyak K, Kato JY, Solomon MJ, Sherr CJ, Massague J, Roberts JM and Koff A (1994*b*) p27Kip1, a cyclin-Cdk inhibitor, links transforming growth factor-beta and contact inhibition to cell cycle arrest. *Genes Dev* **8**: 9–22
Porter PL, Malone KE, Heagerty PJ, Alexander GM, Gatti LA, Firpo EJ, Daling JR and Roberts JM (1997) Expression of cell-cycle regulators p27Kip1 and cyclin E, alone and in combination, correlate with survival in young breast cancer patients. *Nature Med* **3**: 222–225
Raff MC (1992) Social controls on cell survival and cell death. *Nature* **356**: 397–400
Reynisdottir I, Polyak K, Iavarone A and Massague J (1995) Kip/Cip and Ink4 Cdk inhibitors cooperate to induce cell cycle arrest in response to TGF-beta. *Genes Dev* **9**: 1831–1845
Robertson J (1997) Prognostic and response markers in the management of breast cancer. *Cancer Treatment Rev* **23**: S41–48
Saez S, Clark GM and McGuire WL (1988) Prognostic factors in cancer of the breast. *Clin Oncol* **2**: 103–115
Shao Z-M, Jiang M, Wu J, Han Q-X, Zhang T-Q, Shen Z-Z and Fontana JA (1996) Identification of spontaneous programmed cell death during development of human breast cancer. *Oncology Rep* **3**: 1133–1136
Tan P, Cady B, Wanner M, Worland P, Cukor B, Magi-Galluzzi C, Lavin P, Draetta G, Pagano M and Loda M (1997) The cell cycle inhibitor p27 is an independent prognostic marker in small (T1a,b) invasive breast carcinomas. *Cancer Res* **57**: 1259–1263
Tomei LD, Kanter P and Wenner CE (1988) Inhibition of radiation-induced apoptosis in vitro by tumor promoters. *Biochem Biophys Res Commun* **155**: 324–331
Toyoshima H and Hunter T (1994) p27, a novel inhibitor of G1 cyclin-Cdk protein kinase activity, is related to p21. *Cell* **78**: 67–74
Wage S, Hannon GJ, Beach D and Stillman B (1994) The p21 inhibitor of cyclin-dependent kinases controls DNA replication by interaction with PCNA. *Nature* (Lond) **369**: 574–578

 *© Cancer Research Campaign 1999*
